# Editorial on Special Issue “Hydrogels for Biomedical Applications: New Knowledge”

**DOI:** 10.3390/gels8020080

**Published:** 2022-01-28

**Authors:** Naphtali O’Connor

**Affiliations:** 1 Department of Chemistry, Lehman College, City University of New York, Bronx, NY 10468, USA; NAPHTALI.OCONNOR@lehman.cuny.edu; 2 Ph.D. Programs in Chemistry and Biochemistry, The Graduate Center, City University of New York, New York, NY 10016, USA

Hydrogels are a network of hydrophilic polymers or lower molecular weight gelators capable of retaining a large quantity of water within three-dimensional networks without dissolving. In Otto Wichterle’s 1960 Nature publication titled “Hydrophilic Gels for Biological Use”, he proposed the use of pHEMA hydrogels for soft contact lenses and other biomedical applications. Since then, the utilization of hydrogels has grown into well-developed commercial and research arenas. Hydrogels are now commercially available as soft contact lenses, wound dressings and diaper absorbents, to name a few. Current hydrogel research holds the promise of biomedical advances that include controlled drug delivery, biosensors, tissue engineering and regenerative medicine. Exciting new methods are being explored to deploy hydrogels in these applications such as injectable hydrogels, electrospinning and bioprinting. Some of these applications are highlighted in this Special Issue.

A common strategy to develop hydrogels for biomedical applications is to use naturally derived materials to impart biocompatibility or biological activity. In their article, M. Zahid et al. incorporated seed extracts from the Persian traditional medicinal plant *Raphanus sativus* L. into alginate hydrogels [1]. The plant is known to possess antioxidant, antimicrobial and anti-inflammatory properties, and its inclusion in hydrogels showed beneficial wound healing properties. In the same spirit, N. O’Connor et al. utilized naturally derived polydopamine to prepare wound-healing hydrogels [2]. Polydopamine was prepared by polymerizing the nuerotransmitter dopamine and after crosslinking to form hydrogels resulted in materials with antioxidant activity that exhibited significant cell migration in wound healing assays. The authors N. Zerbinati et al. examined a commerial hyaluronic acid hydrogel as a restorative for mesotherapy [3]. Mesotherapy is a non-surgical technique that uses micro-injections to revitalize skin or hair. In vitro evaluations with keratinocytes showed elevated angiogenesis and protection against oxidative stress. The contribution by G. Stabile et al. also examined a commercial gel but to combat vaginal dryness experienced by post-menopausal women [4]. The authors developed an in vitro skin model of dryness using reconstituted vaginal tissue.

To expand the application of hydrogels as biomaterials there is the continued need to develop new materials. With growing interest in injectable hydrogels as scaffolds or to deliver therapeutics, low molecular weight gelators have attracted increased attention. Low molecular weight gelators form hydrogels through high ordered structures bound by intermolecular interactions such as hydrogen bonding and π–π stacking. An example of this can be seen in the contribution by A. Croitoriu et al., where they used FMOC-protected amino acids to assemble supramolecular gels [5]. The authors C. Fiorica et al. examined the physiochemical properties of low molecular weight gellan gum products resulting from hydrolysis of the high molecular weight biopolymer [6]. Gellan gum is a high molecular polyscaharide that is widely used as a biomaterial, but it can require high temperatures for aqueous dissolution. This makes its use for many biomedical applications difficult. The authors found that considerably lower molecular weight gellan gum products provided similar physicochemal properties without the processing down sides of the much higher molecular weight parent polymer.
